# Coffee Silverskin Extract Protects against Accelerated Aging Caused by Oxidative Agents

**DOI:** 10.3390/molecules21060721

**Published:** 2016-06-01

**Authors:** Amaia Iriondo-DeHond, Patricia Martorell, Salvador Genovés, Daniel Ramón, Konstantinos Stamatakis, Manuel Fresno, Antonio Molina, Maria Dolores del Castillo

**Affiliations:** 1 Institute of Food Science Research (CSIC-UAM), Nicolás Cabrera 9, 28049 Madrid, Spain; amaia.iriondo@csic.es; 2 Biópolis SL, Parc Científic Universitat de Valencia, Catedrático Agustín Escardino 9, edificio 2, 46980 Valencia, Spain; patricia.martorell@biopolis.es (P.M.); salvador.genoves@biopolis.es (S.G.); daniel.ramon@biopolis.es (D.R.); 3 Centro de Biología Molecular Severo Ochoa (CBM-SO) (CSIC-UAM), Nicolás Cabrera, 1, 28049 Madrid, Spain; kstamatakis@cbm.csic.es (K.S.); mfresno@cbm.csic.es (M.F.); 4 Beacon Biomedicine, Parque Científico de Madrid, Santiago Grisolia 2, D130/L145, 28760 Madrid, Spain; amolina@advmedprojects.com

**Keywords:** coffee silverskin, oxidative stress, UVC radiation, chlorogenic acid, skin health, accelerated aging, nutricosmetic, dermaceutic

## Abstract

Nowadays, coffee beans are almost exclusively used for the preparation of the beverage. The sustainability of coffee production can be achieved introducing new applications for the valorization of coffee by-products. Coffee silverskin is the by-product generated during roasting, and because of its powerful antioxidant capacity, coffee silverskin aqueous extract (CSE) may be used for other applications, such as antiaging cosmetics and dermaceutics. This study aims to contribute to the coffee sector’s sustainability through the application of CSE to preserve skin health. Preclinical data regarding the antiaging properties of CSE employing human keratinocytes and *Caenorhabditis elegans* are collected during the present study. Accelerated aging was induced by *tert*-butyl hydroperoxide (*t*-BOOH) in HaCaT cells and by ultraviolet radiation C (UVC) in *C. elegans*. Results suggest that the tested concentrations of coffee extracts were not cytotoxic, and CSE 1 mg/mL gave resistance to skin cells when oxidative damage was induced by *t*-BOOH. On the other hand, nematodes treated with CSE (1 mg/mL) showed a significant increased longevity compared to those cultured on a standard diet. In conclusion, our results support the antiaging properties of the CSE and its great potential for improving skin health due to its antioxidant character associated with phenols among other bioactive compounds present in the botanical material.

## 1. Introduction

**Figure 1:**
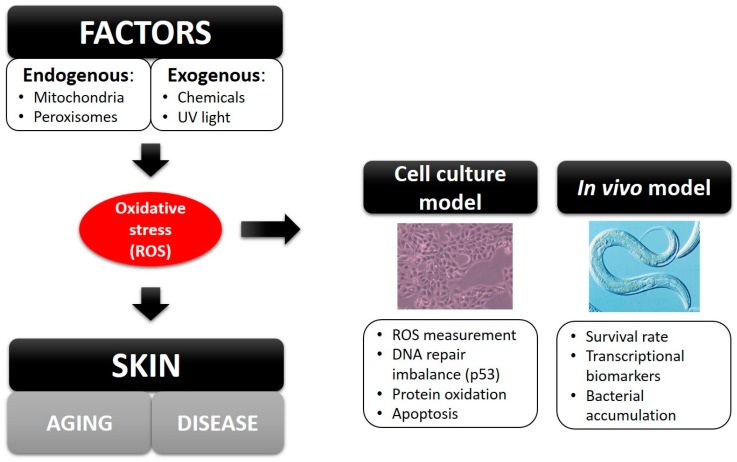
Biomarkers used in cell culture models or *in vivo* models to study the effects of oxidative stress.

Oxidative stress is a major cause of skin accelerated aging and diseases, which is defined as the imbalance between reactive oxygen species (ROS) and antioxidants. Generally, cells are able to balance the production of oxidants and antioxidants. However, when cells are subjected to excessive levels of ROS or as a result of antioxidant depletion, oxidative stress occurs [1]. Under normal conditions, ROS are natural byproducts produced in mitochondria, the peroxisome and the plasma membrane, which have positive physiological effects on cells, such as killing microorganisms, acting as a second messenger in cellular differentiation and proliferation and regulating signal transduction [2]. However, ROS can also be generated by exogenous sources (UV radiation or chemical agents) and cause DNA, protein and lipid damage. This can lead to skin diseases, such as dermatitis, sunburn, acne, eczema, vasculitis, psoriasis and cancer [3]. Damage caused by oxidative stress can be studied in *ex vivo* models [4] or *in vivo* models [5] using the biomarkers described in Figure 1.

Coffee beverage is known for the antioxidant properties of its components, such as caffeine, chlorogenic acid (CGA), hydroxycinnamic acids and melanoidins [6]. In the preparation of this beverage, over 90% of the raw material is discarded as an agricultural by-product. The valorization of such wastes using the biorefinery approach represents a real contribution of many industries for sustainable and competitive development [7].

Many plant extracts and natural compounds are emerging as candidates for the protection of the effects of UV-induced damage on skin; for instance, resveratrol [8,9], citrus and rosemary extract [10] or *Castanea sativa* extract [11]. Various studies suggest that coffee extracts can protect skin cells against photoaging induced by UV irradiation, as well [12,13,14]. In this context, the biomass resulting from coffee roasting (coffee silverskin) could go through biorefinery processes for use as a bioactive compound as a “dermaceutical” in cosmetics. The interest of using coffee silverskin aqueous extract (CSE) in cosmetics was proposed for the first time by del Castillo *et al.* in a patent application filed in 2011, which became public in 2013 (WO/2013/004873) [15]. Very recently, Rodrigues *et al.* tested the *in vitro* antioxidant and antimicrobial capacity of CSE and its cytotoxicity in human skin cells [16]. However, CSE’s ability to protect from sun radiation has not been studied yet. Health benefits of CSE have been associated with its complex and particular chemical composition in bioactive compounds, such as chlorogenic acid (CGA), caffeine, melanoidins and dietary fiber, among others [15,17].

A standard keratinocyte cell culture monolayer can be used to simulate the physiology of the epidermal layer of skin [18]. Such human-derived *in vitro* models are of extreme value for the study of the potential health effects of bioactive compounds on skin when used by topical administration [19]. To the best of our knowledge, the potential of aqueous CSE to reinforce the antioxidant defense of human skin cells has not been previously reported, and it is one of the main goals of the present study. However, since cells grown in monolayers cannot capture the relevant complexity of the *in vivo* microenvironment [19], it is interesting to study the effect of such compounds *in vivo*.

Many biological processes are conserved between humans and *C. elegans*. This nematode has been widely used in aging studies for two reasons: it is a multicellular organism with a fully-sequenced genome, and it has a short lifespan. This nematode is also revealed to have evolutionarily-conserved pathways for aging [20]. In this context, *C. elegans* is the ideal model, since it combines topical and oral antioxidant administration, which is the favored recommendation [21]. Additionally, *C. elegans* is becoming a fast and inexpensive *in vivo* tool for the cosmetic and pharmaceutical industries for compound screening. There is no ethical problem in the use of *C. elegans*, as this nematode is not regarded as an animal in the EU regulation (Directive 2010/63/EU), and the results obtained are consistent with higher animal models, which enable subsequent pre-clinical and clinical trials to be more oriented. No previous studies on the nutricosmetic antiaging effect of CSE using animal models have been published.

The aim of this study is to evaluate the feasibility of CSE to preserve skin health and to reduce the risk of accelerated aging and skin diseases due to oxidative stress induced by physical and chemical agents. The final intention of the investigation is to contribute to the coffee sector’s sustainability through the implementation of the biorefinery concept. Preclinical data regarding the antiaging properties of CSE employing human keratinocytes (HaCaT cells) and *Caenorhabditis elegans* as the animal model are collected during the present study. To do this, accelerated aging was induced by *t*-BOOH (0.5 mM) in HaCaT cells and by UVC in *C. elegans*.

## 2. Results

### 2.1. Study of Coffee Silverskin in HaCaT Cells

Prior to the evaluation of the effect of CSE on cells, we first evaluated the *in vitro* antioxidant capacity of the CSE by the ABTS^•+^ radical cation decolorization assay. An overall antioxidant capacity value of 319.3 CGA equivalents (µmol)/gram of CSE and an IC_50_ value of 373.4 μg/mL were obtained for the trapping capacity of cationic free radicals of CSE (Figure S1, Supplementary Materials). These results demonstrate that the patented CSE possesses *in vitro* antioxidant properties.

Then, we determined the cytotoxic effect of CSE (0.01 mg/mL, 0.1 mg/mL 0.5 mg/mL and 1 mg/mL), CGA (6.88 µg/mL), caffeine (19.86 µg/mL) and vitamin C (0.1 µg/mL) on HaCaT cells using the 3-(4,5-dimethylthiazole-y)-2,5-diphenyltetrazolium (MTT) assay. The concentrations of CGA and caffeine used in this study are equivalent to those present in 1 mg/mL of CSE [22]. No significant decrease (*p* > 0.05) of absorbance was observed after incubation of the compounds when cell viability was measured (Figure S2, Supplementary Materials). These results suggest that CSE, CGA, caffeine and vitamin C at the concentrations tested in this investigation have no adverse effects on the viability of HaCaT cells.

**Figure 2:**
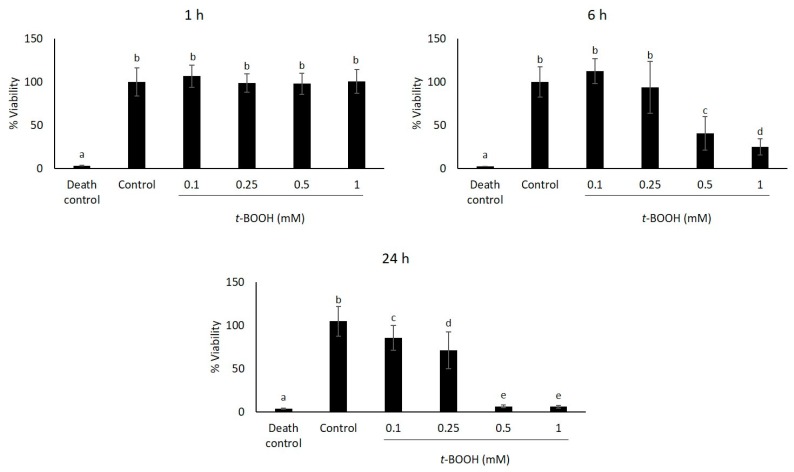
Cell viability determined by the MTT assay of HaCaT cells exposed to different concentrations of *t*-BOOH. Triton X-100 (10%) was used as the death control. Absorbance was measured after 24 h of *t*-BOOH exposure. Data are expressed as the mean of 18 replicates ± SD. Treatments with different letters differ significantly (Tukey test, *p* < 0.05).

In order to study the response of HaCaT cells to oxidative treatment, we initially determined their sensitivity to increasing concentrations of *t*-BOOH by measuring cell viability using the MTT assay (Figure 2). Therefore, cells were treated with different concentrations of *t*-BOOH used previously by Kučera *et al.* (2014) [23] (0.1 mM, 0.25 mM, 0.5 mM and 1 mM), and viability was estimated after 1, 6 and 24 h. The obtained results showed no significant cell viability reduction (*p* > 0.05) when *t*-BOOH was added for one hour. However, cell viability decreased following treatment with *t*-BOOH for 6 and 24 h in a dose-dependent manner. The lowest concentration of *t*-BOOH (0.1 mM) did not reduce cell viability; however, higher concentrations of *t*-BOOH (0.25 mM, 0.5 mM and 1 mM) were cytotoxic to HaCaT cells, since cell viability was significantly reduced (*p* < 0.05) (Figure 2).

Since the *t*-BOOH concentration of 0.5 mM at 6 h caused a significant decrease (*p* < 0.05) in the cell viability of nearly 60% (Figure 2), we decided to choose this concentration to induce oxidative stress in the following experiments.

**Figure 3:**
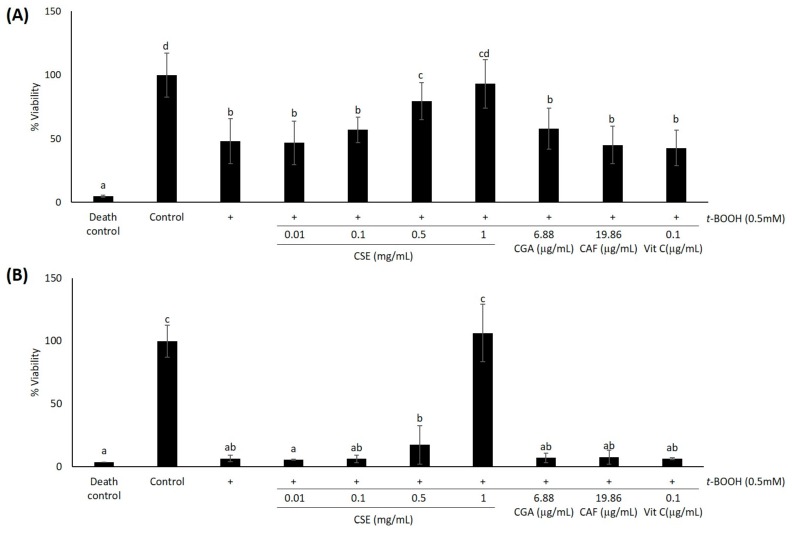
Effect of coffee silverskin extract (CSE), chlorogenic acid (CGA), caffeine (CAF) and vitamin C (Vit C) against oxidative damage induced by *t*-BOOH 0.5 mM. Cells were treated with 0.01–1 mg/mL CSE, 6.88 μg/mL of CGA, 19.86 μg/mL of CAF and 0.1 μg/mL of Vit C for 24 h and further exposed to 0.5 mM *t*-BOOH for 6 h (**A**) or 24 h (**B**). Triton X-100 (10%) was used as the death control. Then, cell viability was measured using the MTT assay. Data represent means ± SD of 18 samples per condition. Different letters denote statistically-significant differences between all treatments (*p* < 0.05).

As we were concerned about the combined effects of *t*-BOOH and CSE on HaCaT cytotoxicity, cells were pre-treated with various doses of CSE for 24 h prior to the induction of oxidative stress with 0.5 mM *t*-BOOH. For cell viability determinations, after pre-treatment with different concentrations of CSE (0.01 mg/mL, 0.1 mg/mL, 0.5 mg/mL and 1 mg/mL), keratinocytes were exposed to 0.5 mM *t*-BOOH for 6 and 24 h. After 6 h of oxidative damage, cell viability decreased significantly (*p* < 0.05) by nearly 60%, in line with the observed results of previous experiments (Figure 3A). When cells were pre-treated with CSE for 24 h prior to oxidation, cell death was diminished when the extract doses used were 0.5 mg/mL and 1 mg/mL. Since there is no significant difference (*p* > 0.05) between control cells and cells pre-treated with 1 mg/mL of CSE, we can suggest that this dose of CSE fully protected cells from death induced by oxidative stress (Figure 3A).

In order to find out if CSE is able to protect cells when oxidation takes place during 24 h, we performed the MTT assay after 24 h of *t*-BOOH 0.5 mM incubation (Figure 3B). In this case, *t*-BOOH-induced oxidation reduced cell viability in a similar way as the death control (*p* > 0.05). Pre-treatment with 1 mg/mL of CSE was the only dose able to protect cells from such extreme cellular damage. No significant differences (*p* > 0.05) were found between control cells and cells pre-treated with 1 mg/mL of CSE. In none of the cases did CGA, caffeine at concentrations equivalent to those present in 1 mg/mL of CSE and vitamin C have a significant effect in the prevention of *t*-BOOH-induced cell death.

**Figure 4:**
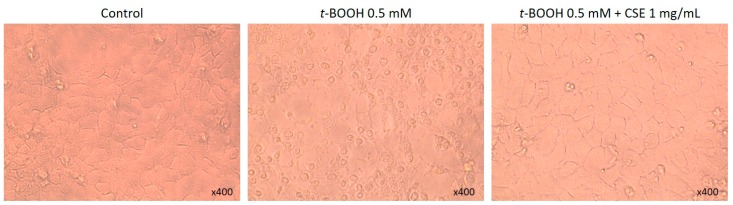
Representative microscopy images (×40) of HaCaT cells after different treatments. Control = untreated cells; *t*-BOOH 0.5 mM = cells treated with 0.5 mM *t*-BOOH for 24 h; *t*-BOOH 0.5 mM; CSE 1 mg/mL = cells pre-treated with 1 mg/mL CSE for 24 h and further exposed to 0.5 mM *t*-BOOH for 24 h.

Figure 4 illustrates the effect of the CSE on the appearance of the HaCaT cell monolayer. *t*-BOOH treatment led to morphological changes, such as cell shrinkage related to cell death. However, pre-treatment with CSE 1 mg/mL prevented these morphological alterations.

**Figure 5:**
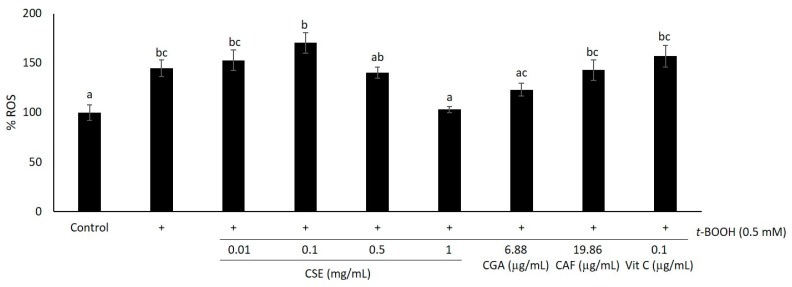
Effect of CSE (0.01–1 mg/mL), CGA (6.88 μg/mL), CAF (19.86 μg/mL) and Vit C (0.1 μg/mL) against oxidative damage induced by *t*-BOOH 0.5 mM. Cells were pre-treated with CSE, CGA, CAF and Vit C for 24 h, incubated with the 2′,7′-dichloro-dihydro-fluorescein diacetate (DCFH-DA) probe for 30 min and further exposed to 0.5 mM *t*-BOOH for 1 h. Then, the fluorescence of intracellular ROS was measured. Data represent the means ± SEM of 18 samples per condition. Different letters denote statistically-significant differences between all treatments (*p* < 0.05).

Considering the prevention of ROS generation, HaCaT cells were incubated with *t*-BOOH 0.5 mM for 1 h, and then intracellular ROS were measured using the 2′,7′-dichloro-dihydro-fluorescein diacetate (DCFH-DA) probe (Figure 5). When *t*-BOOH 0.5 mM was added, intracellular ROS significantly increased (*p* < 0.05) from physiological ROS (100%) to 150% approximately. However, when cells were pre-treated with 1 mg/mL of CSE, ROS were diminished to physiological levels (*p* > 0.05). Neither lower concentrations of CSE nor CGA, CAF at concentrations equivalent to those present in 1 mg/mL of CSE and Vit C had an effect in the prevention of oxidative stress, since no significant differences were found between them and non-pre-treated cells (*p* > 0.05). Taking into account the obtained results from the cell culture experiments, HaCaT cells pre-treated with CSE exhibited a marked resistance to *t*-BOOH-induced oxidative damage (Figure 3, Figure 4 and Figure 5).

### 2.2. Study of Coffee Silverskin in C. elegans

In order to study the *in vivo* antiaging effect of CSE, *C. elegans* under UVC-induced oxidative stress was fed on different concentrations of CSE (0.01 mg/mL, 0.1 mg/mL and 1 mg/mL), CGA (0.1 µg/mL) and vitamin C (0.1 µg/mL) as controls. *C. elegans* has been widely used in many studies as a model to determine the benefits of different compounds and plant extracts on aging-related parameters. In this case, we used wild-type nematodes (N2 strain) grown in nematode growth (NG) medium with CSE.

**Figure 6:**
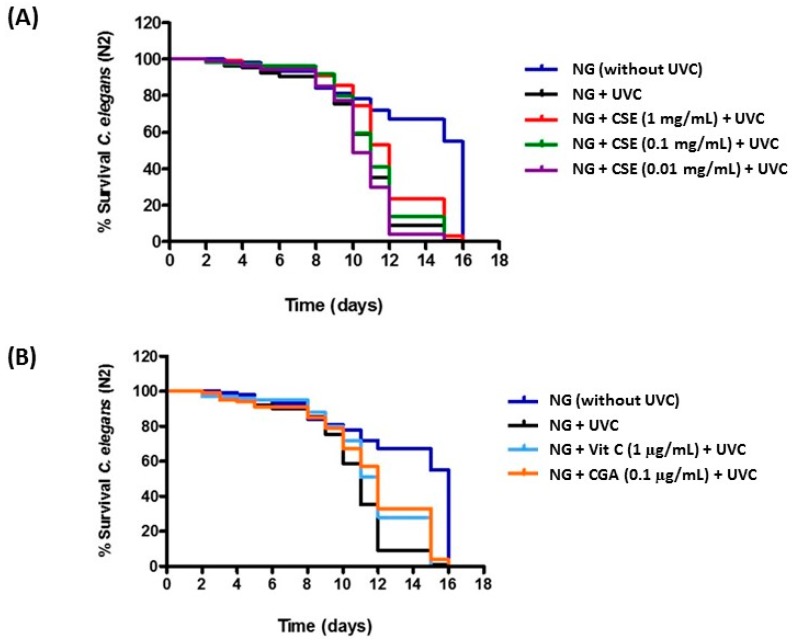
Survival curves of *C. elegans* wild-type strain N2, growing in NG medium supplemented with CGA (0.1 µg/mL) and vitamin C (0.1 µg/mL), as positive controls (**A**) and with CSE (1 mg/mL, 0.1 mg/mL and 0.01 mg/mL) (**B**). Nematodes were treated daily with UVC. A control condition NG without UVC treatment was included. Experiments were performed in triplicate.

With regard to the *in vivo* experiments, CSE was studied in the same concentrations used on HaCaT cells. Nematodes were subjected to a daily UVC treatment (45 s/day) to induce oxidative damage. Thus, UVC treatment provoked a dramatic viability decrease in nematodes as compared to the control conditions (Nematode growth medium without UVC treatment) (Figure 6A,B). This is in accordance with previous reports suggesting an accumulation of DNA damage and a drop of the worm’s survival during chronic UV exposition [24]. Moreover, under UVC treatment, it was determined that the dose of 1 mg/mL of CSE showed an increase in nematodes’ longevity compared to the control conditions (Figure 6A), and in a similar way as vitamin C and CGA used as positive controls (Figure 6B, Table 1). In fact, this increase proved to be significant with a confidence level of 99% (Table 1). These results suggest the protective effect of CSE on the oxidative stress produced by UVC radiation. In relation to the lower doses of CSE, no effect on longevity was observed (Figure 6A, Table 1).

## 3. Discussion

The aim of this study was to obtain novel information regarding the use of CSE and its bioactive compounds, CGA and caffeine, on accelerated aging and skin damage induced by oxidative stress. For this purpose, we used an established human cell culture line (HaCaT cells) as a skin model and *C. elegans* as an animal model.

In our study, values of the overall antioxidant capacity of CSE agree with those reported by Mesías *et al.* [25] and Fernandez-Gomez *et al.* [26]. Results demonstrate that our CSE has powerful antioxidant capacity, in a similar way to other CSE reported by Narita and Inouye [27], del Castillo *et al.* [15] and Borrelli *et al.* [28]. CSE antioxidant capacity may be explained by the presence of polyphenolic compounds, such as chlorogenic acid and melanoidins formed during roasting [28]. It has been suggested that the main antioxidant compounds present in the CSE are CGAs, melanoidins and antioxidant fiber. Such an antioxidant capacity that CSE possesses suggests that it could be used as a good source of bioactive compounds with putative health benefits [17].

Studies using different cell types, such as pancreatic cells, have demonstrated that CSE is not cytotoxic when used at the determined concentrations [26]. However, there are very few studies that report the effects of an aqueous CSE in the HaCaT cell line. Actually, Rodrigues and colleagues are the only ones who have previously studied the effect of CSE in HaCaT cells and fibroblasts, and CSE was not cytotoxic, as well [16,29]. Their studies on human skin cells involved aqueous, hydroalcoholic and ethanolic CSE in a final concentration range of 0.1–1000 µg/mL, the same concentrations used in our study. None of the extracts resulted in being cytotoxic in these cell lines [16]. 

Due to keratinocytes’ location in the human body, these cells are continuously exposed to endogenous and environmental pro-oxidant agents, which increase intracellular levels of reactive oxygen species [30]. When skin is exposed to UV radiation, distinct response pathways are activated. As UV radiation causes the generation of ROS [31], we decided to induce cellular stress with *t*-BOOH. *tert*-Butyl hydroperoxide is a membrane-permeant oxidant that has been extensively used as a model of oxidative stress in different systems [32]. The range of *t*-BOOH doses used in our studies to induce cytotoxicity (0–1 mM) was similar to the one that Alía and colleagues used in HepG2 cells [33].

To investigate the potential of CSE in the protection against oxidative damage, we used effective doses of CSE on an *ex vivo* and on an *in vivo* model, HaCaT cells and *C. elegans*, respectively. The spontaneously immortalized human keratinocyte line, HaCaT, is one of the most frequently-used keratinocyte cell lines because of its highly preserved differentiation capacity [18]. HaCaT cells were pre-treated for 24 h to simulate a chronic use of CSE prior to oxidative damage. Our results suggest that this chronic application of CSE on human skin cells could prevent the effects produced by oxidative stress damage. Since 1 mg/mL of CSE prevented from cellular death induced by 0.5 mM *t*-BOOH and was able to reduce induced intracellular ROS to physiological levels (Figure 3, Figure 4 and Figure 5), we could use this extract in this concentration to preserve skin health. Other studies have shown that CSE is able to protect pancreatic cells from induced oxidative damage [22]. Furthermore, there are other compounds present in coffee silverskin that can protect from UV-induced photodamage. On the one hand, research demonstrates that caffeine inhibited the development of squamous cell carcinomas when mice were previously treated with UV radiation. This suggests that caffeine is able to absorb as an additional sunscreen in the UV range and to prevent photodamage and photocarcinogenesis [34]. On the other hand, another study showed how *Coffea arabica* leaf extract and its constituents, chlorogenic acid and caffeic acid, diminished UV-induced photoaging by inhibiting MMPs through ROS scavenging and down-regulation of the MAP kinase pathway [12]. 

The prevention of UV damage is one of the most effective ways of diminishing the effects of photoaging, one of the biggest factors contributing to facial wrinkles [12]. The use of nematodes in this study is an interesting way of combining topical and oral administration of the bioactive compounds present in CSE. In fact, many studies suggest that using a combination of topical and oral antioxidants provides better results in the protection from UV radiation [21,35].

In the present study, we showed that UV-induced oxidative stress significantly decreased the viability and the lifespan of *C. elegans*. Furthermore, CSE restored the lifespan of oxidative stress-UV-induced *C. elegans*. It is well known that UV radiation is the main cause of photoaging and induces cell and tissue damage as the production of ROS, which leads to DNA damage [36]. In this sense, CSE could be reducing the oxidative stress accumulation and, therefore, the DNA damage, as previously demonstrated with other antioxidant compounds, such as tocotrienol [37]. Although previous reports have been made about the functional properties of coffee in *C. elegans* [38,39], we report for the first time the potential of a natural extract from coffee silverskin by-product for UV radiation protection, which could be very interesting for dermo- and nutria-cosmetic companies developing new products targeting photoaging. The chemical composition of coffee extracts studied by other authors is different from that corresponding to the coffee silverskin extract hereby investigated and patented by our research group.

Other studies use this nematode to study the effect of plant extracts on its lifespan. There are other plant extracts containing CGA and other polyphenols able to exert an antiaging effect on *C. elegans.* For instance, crude blueberry extract and blueberry polyphenols (including an hydroxycinnamic ester fraction containing CGA) have lengthened the nematode’s mean lifespan by 28% [40]. Moreover, Vayndorf *et al.* observed that when *C. elegans* was pre-treated with whole apple extracts, worms were more resistant to stresses, such as heat, UV radiation and pathogenic infection, suggesting that cellular defense and immune system functions were improved. The authors suggest a possible antioxidant mechanism underlying the antiaging effects of whole apple phytochemicals [41]. In addition, polydatin, a natural resveratrol glycoside, was found to significantly extend the mean lifespan of worms by up to 30.7% and 62.1% under normal and heavy metal-induced acute stress conditions, respectively [42]. Some of these extracts have already shown their effectiveness as antiaging agents in humans [43], validating the feasibility of the animal model for the acquisition of preclinical data on the nutraceutical benefits of botanicals.

The antioxidant capacity of CSE is due to phenolic compounds, such as free chlorogenic acids and its derivatives, among others. Since in the cell culture model, neither CGA nor CAF at concentrations equivalent to those present in 1 mg/mL of CSE were able to prevent from oxidative damage, it seems that CGA and CAF are not solely responsible for the antioxidant capacity of CSE found under our particular experimental conditions. In fact, there are other antioxidant compounds present in the sample, such as melanoidins formed during roasting and antioxidant fiber, that may also contribute to such an effect [17,25]. Further research is needed to identify those compounds responsible for the CSE cellular antioxidant effect. Such a property may be due to a synergic effect derived from the combination of the bioactive compounds present in CSE.

The results obtained in the present study support the feasibility of using coffee silverskin extract in skin care for protection against skin diseases associated with oxidative stress and accelerated aging induced by UV radiation. The application of the extracts in cosmetology and dermatology represent an opportunity to increase the sustainability and competiveness of the coffee sector. The obtained data support that coffee is not only for drinking, in agreement with data reported by others indicating the feasibility of applying the biorefinery concept to the coffee sector [44].

Apart from roasting to prepare the coffee brew, the best known application for green coffee is as a natural source of antioxidants [45] and as weight-loss supplements [46]. Furthermore, *C. arabica* green coffee beans present a high content of oil, wax and unsaturated fatty acids, which leads to a high sun protection factor [47]. Coffee silverskin has also been suggested for use in cosmetic care products [15,16,48]. However, very little is known about the contribution of the individual components of the extracts to this effect and their mechanism of action. There is a lack of information regarding the chemical composition of the silverskin extract, although it is of great interest. Because of the accepted safety profile of these compounds, the addition of coffee extracts to sunscreen products could be considered [34]. Del Castillo *et al.* proposed the use of coffee silverskin in skin care cosmetics to prevent physiological aging in 2011 [15]. Last year, Rodrigues and colleagues studied a hand cream formulation containing 2.5% (*w*/*w*) of CSE. Their studies confirm that it is possible to include CSE in a hand cream formulation and that such a product is stable under extreme conditions and safe for topical use [29].

Results support that the patented CSE (WO/2013/004873) feasibly reduces the production of intracellular ROS in keratinocytes, improving skin health. Additionally, CSE protects against photoaging induced by UV radiation. 

## 4. Materials and Methods

### 4.1. Materials

Chlorogenic acid, 2,2′-azino-bis (3-ethylbenzothiazoline-6-sulphonic acid) diammonium salt (ABTS), caffeine, ascorbic acid (vitamin C), tert-butyl hydroperoxide (*t*-BOOH), dimethyl sulfoxide (DMSO), 3-(4,5-dimethylthiazole-y)-2,5-diphenyltetrazolium bromide (MTT) and 2′,7′-dichloro-dihydro-fluorescein diacetate (DCFH-DA) were purchased from Sigma Chemical (Sigma-Aldrich, St Louis, MO, USA). Dulbecco’s Modified Eagle's Medium (DMEM) was purchased from Lonza (Basel, Switzerland).

### 4.2. Preparation of Soluble Extracts from Coffee Silverskin

Arabica CSE was produced as described in the patent WO 2013/004873 [15]. Briefly, 50 mg of coffee silverskin were added per H_2_O milliliter. This mixture was stirred at 250 rpm for 10 min; filtered by Whatman paper No. 4; and the filtrate was freeze-dried. Powdered CSE was prepared in aqueous solution, sterile filtered and added to medium to achieve final concentrations of 0.01 mg/mL, 0.1 mg/mL, 0.5 mg/mL and 1 mg/mL. CSE contained 19.87 ± 2.4 mg caffeine/g dry matter and 6.88 ± 1.77 mg CGA/g dry matter [22]. 

### 4.3. CSE Overall Antioxidant Capacity Assay

The trapping capacity of cationic free radicals was evaluated using the method of radical ABTS^•+^ bleaching described by Re *et al.* 1999 [49] and modified by Oki *et al.* [50] for its use in a microplate. A stock solution of the ABTS^•+^ radical was prepared by chemical oxidation of ABTS (7 mM) in the presence of potassium persulfate (140 mM) at room temperature and in darkness for 16 h. The working solution of the ABTS^•+^ radical was prepared by diluting the stock solution 1:75 (*v/v*) in 5 mM sodium phosphate buffer (pH 7.4) to obtain an absorbance value of 0.7 ± 0.02 at 734 nm. Since CGA is the major antioxidant component in coffee, CGA calibration was used to calculate overall antioxidant capacity. A 1:10 dilution (*v*/*v*) of the CGA pattern was performed, so that the final concentrations of the CGA pattern used were 11.5 μM, 25 μM, 50 μM, 75 μM, 115 μM and 200 μM. Then, 30 μL of the samples and 270 μL of the working solution of ABTS^•+^ radical were placed in a microplate (Microtest PS plate 96, Sarstedt AG & Co, Nümbrecht, Germany), and absorbance was measured at 734 nm after 10 min in a BioTek plate reader powerWave™ XS (BioTek Instruments, Winooski, VT, USA).

All determinations were carried out in triplicate. Absorbance values were corrected for the solvent, and inhibition percentages were obtained by multiplying the values of ΔA_sample_ by 100.

### 4.4. Cell Culture and Treatments

The HaCaT human keratinocyte cell line was kindly provided by Dr. Miguel Quintanilla (Instituto de Investigaciones Biomédicas “Alberto Sols”, Madrid, Spain). Cells were cultured in Dulbecco’s Modified Eagle’s Medium (DMEM) supplemented with 10% fetal bovine serum (FBS), 1% l-glutamine and 1% penicillin/streptomycin in standard conditions (37 °C, 5% CO_2_, in a humidified incubator, BINDER CB series 2010, Tuttlingen, Germany).

For the treatments with the different compounds, concentrations of CSE (0.01, 0.1, 0.5 and 1 mg/mL), CGA (6.88 μg/mL), caffeine (19.86 μg/mL) and vitamin C (0.1 μg/mL) diluted in DMEM culture medium and filtered through a 0.45-μm membrane were added to cell plates during 24 h. In order to induce oxidative stress in cells, *t*-BOOH was dissolved in MilliQ·H_2_O and added to cell plates during different periods of time (1, 6 and 24 h) and at concentrations ranging from 0.1–1 mM.

### 4.5. Cell Viability Assays

The effect of different concentrations of CSE, chlorogenic acid, caffeine and vitamin C alone or in combination with *t*-BOOH on cell viability was measured using the MTT assay [51]. Cells were cultured at a density of 1.0 × 10^4^ cells per well of a 96-well plate for 3 days until cell confluence was achieved. On the one hand, CSE (0.01 mg/mL, 0.1 mg/mL, 0.5 mg/mL and 1 mg/mL), chlorogenic acid (6.88 µg/mL), caffeine (19.86 µg/mL) and vitamin C (0.1 µg/mL) were incubated for 24 h. Triton X-100 (10%) was used as the death control. On the other hand, different concentrations of *t*-BOOH (0.1 mM, 0.25 mM 0.5 mM and 1 mM) were studied for 1 h, 6 h and 24 h. Subsequently, cells were incubated in MTT solution (0.5 mg/mL) for 1 h at 37 °C. The supernatant was then removed; 100 µL of dimethyl sulfoxide (DMSO, Sigma-Aldrich, Tres Cantos, Madrid, Spain) were added; and the optical density at 570 nm was measured using a microplate reader (BioTek Synergy HT Multi-Mode Microplate Reader, Winooski, VT, USA). Experiments were carried out in triplicate (*n* = 6).

### 4.6. ROS Scavenging Assay

Intracellular ROS scavenging assays were performed by measuring the fluorescence intensity of the 2′,7′-dichloro-dihydro-fluorescein diacetate (DCFH-DA) probe, which was proportional to the amount of ROS formed [52]. A 10 mM solution of DCFH-DA was prepared (5 mg in 1 mL DMSO), and a 50-μL aliquot was separated. Then, 800 μL of DMSO were added to the 50 μL solution. Next, after 24 h of extract incubation, cells were pre-loaded with 2.5 μL/well of this last solution for 30 min at 37 °C. After incubation, DCFH will become dichlorofluorescein (DCF) due to intracellular oxidants and will emit fluorescence. Next, the culture medium was removed; cells were washed with PBS; and *t*-BOOH was added for 1 h. Then, fluorescence was measured at 485 nm/528 nm (BioTek Synergy HT Multi-Mode Microplate Reader). Experiments were carried out in triplicate (*n* = 6).

### 4.7. C. elegans Lifespan Assays

To measure *C. elegans* survival rates after exposure to oxidative stress induced by UVC radiation, we employed synchronized *C. elegans* N2 strain eggs. They hatched in NG medium (nematode growth) and were cultured on agar plates containing *Escherichia coli* OP50 strain. After 3 days of growth at 20 °C, worms were transferred to plates containing NG medium, *E. coli* and different concentrations of CSE (0.01 mg/mL, 0.1 mg/mL and 1 mg/mL), CGA (0.1 µg/mL) or vitamin C (0.1 µg/mL). Then, worms (100 worms per treatment) were incubated for 15 days at 20 °C and transferred every 2 days to fresh media plates to score viability. During this period, worms were subjected daily to UVC radiation for 45 s. The animals were scored as dead if they failed to respond to a platinum wire. All assays were performed in triplicate.

### 4.8. Statistical Analyses

Data obtained from *ex vivo* experiments were expressed as the mean ± SD of 18 determinations. One-way analysis of variance (ANOVA) was performed for cytotoxicity and ROS analysis in HaCaT cells. Statistical comparisons of the different treatments were performed using Tukey’s test. Values of *p* < 0.05 were considered statistically significant. All statistical analyses were performed using the R package software environment Version 3.2.0 (http://www.r-project.org/).

Survival curves of the cultured nematodes in the presence of CSE, CGA or vitamin C were plotted and analyzed using GraphPad Prism 4 (http://www.graphpad.com/scientific-software/prism/) to study the significance in the viability increase of *C. elegans* among the different conditions. Values of *p* < 0.05 were considered statistically significant.

## 5. Conclusions

We provide scientific evidence with regard to the antioxidant protective effects of CSE in human skin cells and *in vivo* using *C. elegans*, an experimental model. Pure CGA and CAF at the concentrations equivalent to those present in 1 mg/mL of CSE do not seem to be effective in the protection of HaCaT cells from oxidative damage, so further experiments should be performed in order to determine their contribution to the overall antioxidant effect of the extract. CSE is a complex mixture of antioxidants, including CGA, melanoidins and others. Therefore, the protective effect of CSE may be due to the synergistic combination of individual compounds, including phenols, such as CGA. Additional investigation should be carried out to identify all of the antioxidants forming the food matrix. In conclusion, it can be said that CSE has the potential to be used as an ingredient in skin care products for topical use and as nutricosmetic to prevent accelerated skin aging induced by oxidative stress caused either by chemical of physical agents (photoaging).

## Figures and Tables

**Table 1:** Effect of CSE on *C. elegans* lifespan with UVC treatment.

Strain	Treatment	Life Expectancy (50%) (Days)	*p*-Value
N2	NG	11	
	NG + Vit C (0.1 µg/mL)	12	0.0021 **
	NG + CGA (0.1 µg/mL)	12	0.0003 ***
	NG + CSE (1 mg/mL)	12	0.0013 **
	NG + CSE (0.1 mg/mL)	11	0.3364 (NS)
	NG + CSE (0.01 mg/mL)	10	0.2133 (NS)

NG = nematode growth; Vit C = vitamin C; CGA = chlorogenic acid; CSE = coffee silverskin extract; NS: not significant; ** = *p* < 0.05; *** = *p* < 0.001.

## Supplementary Materials

Supplementary materials can be accessed at: http://www.mdpi.com/1420-3049/21/6/721/s1.
