# Supplementary material for: Coffee Silverskin Extract Protects against Accelerated Aging Caused by Oxidative Agents
Source: Molecules. 2016 Jun 1;21(6):721. doi: 10.3390/molecules21060721 (PMC6274150; doi:10.3390/molecules21060721)
Supplement: Supplementary file 1 [file molecules-21-00721-s001.pdf]

Supplementary Materials: Coffee Silverskin Extract Protects Against Accelerated Aging Caused by Oxidative Agents

Amaia Iriondo-DeHond, Patricia Martorell, Salvador Genovés, Daniel Ramón, Konstantinos Stamatakis, Manuel Fresno, Antonio Molina and María Dolores del Castillo

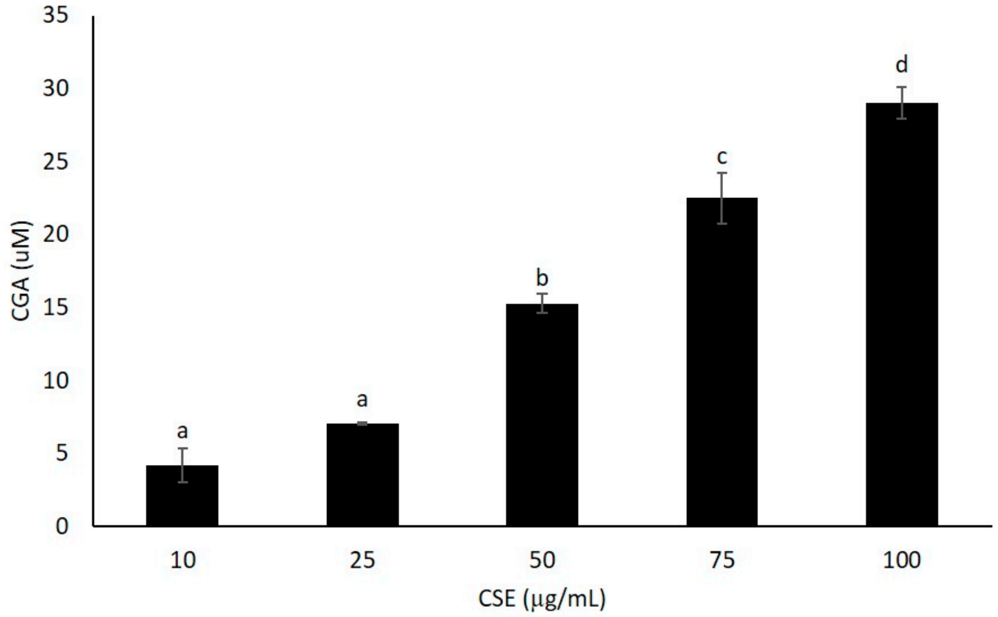

Figure S1. Antioxidant capacity of CSE determined by the ABTS$^{\bullet+}$ assay represented in CGA equivalents (µM). Samples with different letter differ significantly (Tukey Test, $p < 0.05$).

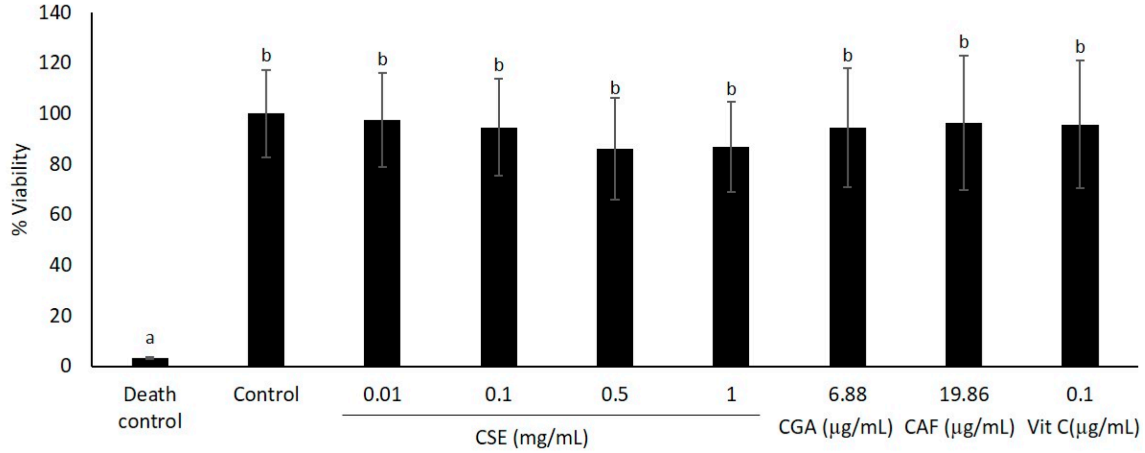

Figure S2. Cytotoxicity assessment of CSE (0.01 mg/mL, 0.1 mg/mL, 0.5 mg/mL and 1 mg/mL), CGA (6.88 µg/mL), caffeine (19.86 µg/mL) and vitamin C (0.1 µg/mL) determined by the MTT method. Triton X-100 (10%) was used as the death control. Results represent the percentage of viable cells after 24 hours of incubation with the different compounds. Data are expressed as the mean of 18 replicates ± SD. Treatments with different letters differ significantly (Tukey Test, $p < 0.05$).
